# Nonvirally Modified Autologous Primary Hepatocytes Correct Diabetes and Prevent Target Organ Injury in a Large Preclinical Model

**DOI:** 10.1371/journal.pone.0001734

**Published:** 2008-03-05

**Authors:** Nelson K. F. Chen, Jen San Wong, Irene H. C. Kee, Siang Hui Lai, Choon Hua Thng, Wai Har Ng, Robert T. H. Ng, Soo Yong Tan, Shu Yen Lee, Mark E. H. Tan, Jaichandran Sivalingam, Pierce K. H. Chow, Oi Lian Kon

**Affiliations:** 1 Division of Medical Sciences, National Cancer Centre, Singapore, Republic of Singapore; 2 Department of General Surgery, Singapore General Hospital, Singapore, Republic of Singapore; 3 Department of Experimental Surgery, Singapore General Hospital, Singapore, Republic of Singapore; 4 Centre for Forensic Medicine, Health Sciences Authority, Singapore, Republic of Singapore; 5 Department of Oncologic Imaging, National Cancer Centre, Singapore, Republic of Singapore; 6 Department of Pathology, Singapore General Hospital, Singapore, Republic of Singapore; 7 Singapore National Eye Centre, Singapore, Republic of Singapore; U.S. Public Health Service, The National Institute of Diabetes and Digestive and Kidney Diseases, National Institutes of Health, United States of America

## Abstract

**Background:**

Current gene- and cell-based therapies have significant limitations which impede widespread clinical application. Taking diabetes mellitus as a paradigm, we have sought to overcome these limitations by *ex vivo* electrotransfer of a nonviral insulin expression vector into primary hepatocytes followed by immediate autologous reimplantation in a preclinical model of diabetes.

**Methods and Results:**

In a single 3-hour procedure, hepatocytes were isolated from a surgically resected liver wedge, electroporated with an insulin expression plasmid *ex vivo* and reimplanted intraparenchymally under ultrasonic guidance into the liver in each of 10 streptozotocin-induced diabetic Yorkshire pigs. The vector was comprised of a bifunctional, glucose-responsive promoter linked to human insulin cDNA. Ambient glucose concentrations appropriately altered human insulin mRNA expression and C-peptide secretion within minutes *in vitro* and *in vivo*. Treated swine showed correction of hyperglycemia, glucose intolerance, dyslipidemia and other metabolic abnormalities for ≥47 weeks. Metabolic correction correlated significantly with the number of hepatocytes implanted. Importantly, we observed no hypoglycemia even under fasting conditions. Direct intrahepatic implantation of hepatocytes did not alter biochemical indices of liver function or induce abnormal hepatic lobular architecture. About 70% of implanted hepatocytes functionally engrafted, appeared histologically normal, retained vector DNA and expressed human insulin for ≥47 weeks. Based on structural tissue analyses and transcriptome data, we showed that early correction of diabetes attenuated and even prevented pathological changes in the eye, kidney, liver and aorta.

**Conclusions:**

We demonstrate that autologous hepatocytes can be efficiently, simply and safely modified by electroporation of a nonviral vector to express, process and secrete insulin durably. This strategy, which achieved significant and sustained therapeutic efficacy in a large preclinical model without adverse effects, warrants consideration for clinical development especially as it could have broader future applications for the treatment of other acquired and inherited diseases for which systemic reconstitution of a specific protein deficiency is critical.

## Introduction

Consistent demonstration that hepatocyte transplantation could correct metabolic abnormalities in experimental animals [1]–[4] led naturally to clinical attempts to treat metabolic disorders about 10 years ago [5]. As of 2006, 21 patients worldwide had received hepatocyte transplantation for liver-directed metabolic disorders with, however, only modest success [6]. Several reasons may explain the incommensurate clinical outcomes of hepatocyte transplantation in human subjects compared to murine models. Metabolic correction in experimental animals is greatly favored under conditions of therapeutic liver repopulation (i.e. when donor hepatocytes have a natural or iatrogenic proliferative advantage over hepatocytes of the recipient host) [7]. However, such conditions rarely occur naturally in humans and, if induced, carry unacceptable clinical risks. Nearly all hepatocyte transplantations have used allogeneic donors. Despite allografts from living-related donors and attempts at optimizing immunosuppresion regimens [8], [9], allograft rejection is another reason for the limited success of hepatocyte transplantation to date. Although conventional gene therapy methods of in vivo vector administration appear to overcome these difficulties, they present other problems. These are the general inability to limit viral vector delivery only to specific cell types and the known risks of immunogenic and genotoxic adverse effects. Moreover, transduced cells that express viral proteins are targets for host immunological destruction–a cause of transient transgene expression [10].

Unlike inborn errors of metabolism in which the liver is the primary disease organ, other inherited and acquired disorders caused by lack of specific proteins e.g. diabetes mellitus and the hemophilias, could benefit more readily from hepatocyte transplantation since liver repopulation is not required to correct these diseases. Moreover, as the liver is structurally and functionally normal, therapeutic autologous hepatocyte transplantation could be feasible. Combining autologous rather than allogeneic hepatocytes with ex vivo gene transfer has several advantages. Hepatocytes from well-planned liver resections are likely to be of higher quality, can be used fresh rather than cryopreserved and allow the use of effective techniques for transfecting primary somatic cells e.g. ex vivo high voltage electroporation that would cause tissue necrosis in vivo. Autologous cells also overcome the problem of donor scarcity and do not subject patients to chronic immunosuppression.

We have chosen insulin-deficient diabetes mellitus as a clinically relevant model to investigate the ability of autologous adult hepatocytes to reconstitute normal metabolism in a large animal model. Although many studies have shown amelioration and even cure of murine diabetes [11]–[15], durable correction has not yet been reported using autologous cells in a preclinical model, indicating that current techniques are not easily scalable for clinical application. Hepatocytes are proficient in protein synthesis and secretion, and are physiologically glucose-sensing cells capable of glucose-stimulated transcription [16]. Unlike pancreatic β cells with which they share a common embryonic origin, hepatocytes do not form secretory granules, which has cast doubts on their suitability to function as insulin-secreting cells [17], [18]. In work previously performed on syngeneic murine primary hepatocytes [19], we showed that high glucose concentration stimulated insulin transcription from a bifunctional promoter in p3MTCHins, consisting of the human metallothionein IIA promoter linked to a carbohydrate response element [20], within 6–10 minutes and secretion within 12 minutes. Basal insulin expression was restored within 30 minutes of removing the glucose stimulus.

These results encouraged us to extend our approach to a large animal model of diabetes in which we would attempt to modify autologous hepatocytes for insulin secretion. While underlying autoimmunity in type 1 diabetes leads to therapeutic failure after islet or whole pancreas transplantation [21], [22], insulin-secreting hepatocytes are not targets for recurrent autoimmune destruction [23]. If successful, this approach could be simpler than islet transplantation, safer than hepatocyte transduction with viral vectors and less empirical than transdifferentiating hepatocytes into β-like cells. Moreover, primary adult hepatocytes would not be expected to have the tumorigenic propensity of β-like cellular implants derived from embryonic stem cells and modified cell lines [24], [25]. Other strategies being developed for cell-based correction of metabolic deficiencies require reliable and efficient differentiation of liver-derived cells or stem cells into the desired cellular phenotype e.g. insulin or clotting factor production. However, directed and trans-differentiation require several weeks of manipulation and culture *in vitro* during which chromosomal aberrations are known to develop [26], [27]. We now describe ex vivo electrotransfer of p3MTCHins into primary hepatocytes freshly isolated from diabetic Yorkshire swine that were autologously reimplanted directly into the liver parenchyma, under visual or ultrasonic guidance, in a single 3-hour procedure. These modified hepatocytes were effective insulin-secreting bioimplants as evidenced by significant and durable correction of hyperglycemia and other metabolic abnormalities associated with diabetes, without evidence of tumor formation for at least 47 weeks. We also showed by morphological studies and transcriptome analyses that target organ injury secondary to poorly controlled diabetes was substantially attenuated in treated swine.

## Methods

### Isolation of Primary Porcine Hepatocytes

A surgically excised liver wedge (50 cm^3^) was sequentially perfused (flow rate 8–10 ml/min) with 2.5 mM EGTA in calcium-free Dulbecco's phosphate buffer (8–10 min) and 0.3% (w/v) collagenase IV-S (Sigma-Aldrich, U S A) (15–20 min) through two catheters, each ligated to a visible vessel. Uncannulated vessels were sutured. The perfused liver was diced and scraped to release the cells. A cellular fraction enriched in viable hepatocytes was obtained as a pellet after centrifugation (150 g, 15 min at 4°C) on a discontinuous Percoll (GE Healthcare) gradient (15–30–45–60%). Cell viability by trypan blue exclusion was 75–90%.

### Gene Transfer

Isolated hepatocytes were electroporated using the Nucleofector™ system (equal volume mixture of solutions #3551 and #3541; program T20; Amaxa Biosystems, Koln, Germany) or with sterile NC solution having the following composition: 19.8 mM KH_2_PO_4_/80.2 mM K_2_HPO_4_/2 mM NaCl, pH 7.6. Freshly prepared 2 mM ATP and 5 mM reduced L-glutathione were added to NC solution just before use. Electroporation with NC solution was performed in a sterile cuvette (4 mm gap; BTX Instrument Division, Harvard Apparatus, U S A) with a single pulse of 1400 V, 70 µs followed immediately by a single pulse of 160 V, 37 ms delivered from a custom-made pulse generator. Eight µg endotoxin-free plasmid DNA (p3MTChins or pEGFP) was added to 4×10^6^ viable hepatocytes in 0.2 ml electroporation solution before electrical pulsing.

### Induction of Insulin Expression In Vitro

Two×10^6^ hepatocytes were cultured on collagen I-coated 35 mm dishes in DMEM-25 mM glucose (supplemented with 10% fetal calf serum, penicillin 10,000 units/ml and streptomycin 10 mg/ml) in 5% CO_2_ at 37°C for at least 16 h. For static induction, DMEM-25 mM glucose was replaced with DMEM containing increasing concentrations of glucose alone or zinc combined with either 2.5 mM or 25 mM glucose. Conditioned media (24 h) of quadruplicate plates were assayed for human insulin. To determine the time course of glucose-stimulated insulin secretion, DMEM-25 mM glucose was replaced with DMEM-2.5 mM glucose 3 h before commencing induction. Replicate plates were then exposed to DMEM-25 mM glucose for 5–90 min. The mean of 2 baseline time points i.e. 10 minutes and immediately before cells were exposed to 25 mM glucose, was taken as the unstimulated value. A parallel series of plates, after exposure to DMEM-25 mM glucose for 90 min, was returned to DMEM-2.5 mM glucose for 5–90 min during the de-induction phase. At every time point during both induction and de-induction phases, quadruplicate plates (replicate plates from each primary hepatocyte preparations) were processed for human insulin assay in the conditioned medium and total cellular RNA isolation (RNeasy Fibrous Tissue kit, Qiagen, Germany).

### Isolation of Tissue RNA

Tissues (kidney, retina, liver and aorta) harvested during planned autopsies were flash frozen and homogenized with mortar and pestle in liquid nitrogen. Total RNA extracted using the guanidine thiocyanate method [28] was stored at −80°C.

### RT-PCR

Semi-quantitative transcript analyses were performed as described previously [19]. Primer sequences used were:

Human insulin (F: 5′ *ttt gtg aac caa cac ctg tgc*; 3′; R: 5′ *ggt tca agg gct tta ttc cat ct* 3′), Human hypoxanthine phosphoribosyltransferase (F: 5′*gga tta cat caa agc act gaa tag* 3′; R: 5′*ggc tta tat cca aca ctt cgt g* 3′), *S. scrofa* insulin (F: 5′*ggc ctt cgt gaa cca gca c* 3′; R: 5′*cca gct cca cgg cac ctg* 3′), *S. scrofa* glucagons (F: 5′*gtt tac cag tga cta cag c* 3′; R: 5′*gtc tct caa att cat cgt ga* 3′), *S. scrofa* somatostatin (F: 5′*gaa ctg gcc aag tac ttc* 3′; R: 5′*gct cca gcc tca ttt cat* 3′), *S. scrofa* pancreatic polypeptide (F: 5′*cct gcg tgg ctc tgt tac tac* 3′; R: 5′*ggt cag cat gtt gat gta tct ac* 3′), *S. scrofa* hypoxanthine phosphoribosyltransferase I (F: 5′*gaa gag cta ctg taa tga cca g* 3′; R: 5′*gcc agt gtc aat tat atc ttc aac* 3′), Ampicillin resistance gene (F: 5′*gca act tta tcc gcc tcc atc* 3′; R: 5′*gca aac tat taa ctg gcg aac ta* 3′).

### Hormone Radioimmunoassays

Porcine C-peptide (PCP), human insulin and C-peptide (HCP) concentrations were quantified by radioimmunoassays (Linco Research, U S A). We added 250 KIU aprotinin (Sigma-Aldrich) to 1 ml whole blood intended for C-peptide assays. HCP assay had no cross reactivity with PCP, human and porcine insulin. PCP assay was <1% cross-reactive with HCP and was not cross-reactive with human and porcine insulin.

### Animals

All animal handling procedures and animal husbandry were conducted in an Association for Assessment and Accreditation of Laboratory Animal Care (AAALAC)-accredited facility. The experimental protocol was approved by the Institutional Animal Care and Use Committee of the Singapore General Hospital. Male and female Yorkshire pigs were fed standard chow twice daily (5% body weight).

### Metabolic and Biochemical Tests

Capillary blood glucose was determined by ear prick and a glucometer (Ascensia ELITE®, Bayer HealthCare, Germany). We drew femoral venous blood periodically for measurements of HCP, PCP and the following clinical serum and blood analytes: fructosamine, urea, creatinine, electrolytes (potassium, sodium and chloride), total protein and albumin, total bilirubin, alkaline phosphatase, alanine aminotransferase (ALT), gamma-glutamyltransferase (GGT), triglycerides, total and HDL cholesterol.

### Streptozotocin-Diabetic Swine

Pigs were fed ammonium chloride (1.5 g/kg body weight in 500 g moistened chow) 16 h before a bolus ear vein injection over 2 min of streptozotocin (STZ; 150 mg/kg body weight; Zanosar®, Pfizer) followed immediately by 50 ml saline flushing [29]. Exogenous insulin injections (Lantus®, Sanofi Aventis; Humulin® R, Eli Lilly) were administered to diabetic pigs only when fasting blood glucose exceeded 11 mM during the 3-day period before surgery to reduce the risk of post-operative sepsis. Insulin was not administered to any animal at any time after hepatocyte implantation.

### Intravenous Glucose Tolerance Test (IVGTT)

We performed IVGTT on 16 h overnight fasted animals. An internal jugular vein was cannulated (7-French; Arrow International, Inc.) under general anesthesia. To establish the baseline, a saline bolus (equal to the volume of glucose solution) was injected over 3 minutes and blood was drawn 10, 20 and 30 min later for blood glucose, plasma HCP and PCP assays. IVGTT was initiated by a bolus glucose injection (1 g/kg; 25% w/v solution) over 3 minutes followed by 30 ml saline injection to flush the line. Three ml of blood was drawn 1, 3, 5, 10, 20, 30, 45, 60 and 90 min after the glucose bolus for the same assays. To avoid possible artifacts caused by hypovolemia, 3 ml heparinized saline was injected after each blood sampling.

### Surgical Procedures

#### (1) Resection of liver wedge

Fourteen diabetic pigs (9 male and 5 female; 17–23 kg) underwent excision of 30% of the left lateral liver lobe (equivalent to <10% of total liver mass) under general anesthesia 10 days post-STZ administration. The left lateral liver lobe was mobilized as previously described [30]. About 30% of the lobe was resected en masse after applying a non-crushing clamp across it. The main portal vein branch in the resected specimen was immediately cannulated and perfused with cold calcium-free Dulbecco's phosphate buffer. Hemostasis was achieved with 5/0 Prolene sutures and diathermy. Surgicel (Johnson & Johnson, U S A) was applied to the resected surface and the abdominal cavity was washed with 5 ml of an antibiotic solution (penicillin 100 mg/ml and ampicillin 100 mg/ml). The abdominal wound was closed with continuous 2/0 Ethilon and the skin with 3/0 subcuticular Vicryl. All surgical sutures were from Ethicon, U S A.

Primary hepatocytes prepared from excised liver tissue were electroporated ex vivo with 2 µg p3MTChins DNA per 10^6^ viable hepatocytes and immediately reimplanted into the liver parenchyma using one of the methods below with comparable metabolic outcomes.

#### (2) Hepatocyte implantation

##### Open method

The abdominal cavity was left open after liver resection while hepatocyte isolation and electroporation were performed. Transfected hepatocytes in suspension were injected under direct vision in up to 5 sites of the left and right central lobes with a 23G needle (3–4 ml/site). Implantation sites were marked with a 5/0 Prolene suture. The abdominal incision was then sutured close. *Percutaneous method*: The abdominal incision was sutured immediately after liver resection. The suspension of transfected hepatocytes was injected percutaneously into 3 sites in the left and right central liver lobes (5–6 ml/site) using a Spinocan® spinal needle (22G, 3.5 inches long; B. Braun Medical Inc., U S A) under real-time ultrasonic guidance (Acuson XP10, U S A). Gelfoam® suspension (1 ml) (Pharmacia & Upjohn, U S A) was injected upon withdrawal of the needle from the liver parenchyma for hemostasis. The number of hepatocytes implanted using either method was 2.0–3.3×10^8^ in 15 ml DMEM.

The entire procedure from wedge excision to hepatocyte implantation was completed in about 3 hours as a single surgical operation.

### Histology

Paraffin-embedded liver sections (4 µm) were immunostained with the following primary antibodies according to manufacturers' recommended protocols: ready diluted monoclonal mouse anti-human insulin (Zymed Laboratories Inc., U S A), monoclonal mouse anti-rat PCNA (1:50 dilution) and monoclonal rabbit anti-human Ki-67 (1:100 dilution) (both from Acris Antibodies GmbH, Germany). The latter two antibodies bind the corresponding porcine antigens. Bound primary antibody was detected with ChemMate™ Envision™ kit (Dako Cytomation, Denmark). Sections were counterstained with hematoxylin (Sigma-Aldrich). Standard histological evaluations were also performed on paraffin sections stained with hematoxylin and eosin or periodic acid Schiff (PAS) reagent, counterstained with hematoxylin.

### Electron Microscopy

#### Scanning electron microscopy (SEM)

Specimens were fixed in 2.5% glutaraldehyde in phosphate-buffered saline for 24 h at 4°C, then post-fixed in 1% OsO_4, _pH 7.4 for 2 h at room temperature, followed by dehydration in a graded ethanol series and drying in a critical point dryer. Preparations were coated with gold for viewing (JSM-5660, JEOL, Japan). *Transmission electron microscopy (TEM)* Specimens were fixed and post-fixed as for SEM. After dehydration, specimens were embedded in epoxy resin (polymerization at 60°C for 24 h). Ultra-thin sections (90–100 nm) were stained with uranyl acetate and lead citrate and viewed in a microscope (JEM-1220, JEOL, Japan) operated at 100 KV. Basement membrane thickness was measured from TEM images of 20 choroidal capillaries (10 measurements per capillary) and 20 renal glomeruli (10 measurements per glomerulus) using a standard grid. The retina and kidney of three animals (healthy control, untreated diabetic and treated diabetic pigs) were compared. All measurements were made independently by three individuals whose data were pooled for analysis.

### Plasmid DNA Quantitation

Total DNA was extracted from paraffinized tissues (Puregene® DNA purification kit; Gentra Systems, U S A). A standard curve was constructed from real-time PCR by spiking 50 ng pig liver DNA with graded amounts of p3MTChins DNA (0, 1, 10, 50, 100, 500, 1,000, 5,000 and 10,000 pg). Primer sequences amplified the ampicillin resistance gene unique to the plasmid. Plasmid DNA detected in DNA of pig liver sections were quantified from the standard curve-derived equation: log DNA = −0.2507 C_t_+5.4122 (triplicate assays, r^2^ = 0.9952).

### Transcriptome Profiling

#### Study design

We transcriptionally profiled kidney, retina, aorta and liver of normal healthy, treated diabetic and untreated diabetic pigs (n = 2 per group). All animals were matched for age, sex and body weight. Treated diabetic and untreated diabetic animals were autopsied 34–38 weeks and 36–42 weeks, respectively, post-implantation. To ensure that the transcriptome profiles were representative of the entire organs/tissues of interest, we homogenized (i) a whole kidney; (ii) all retinal tissue of one eye; (iii) equal segments from the aortic arch, thoracic and abdominal aorta; and (iv) an equal portion from each liver lobe from every pig. Ground tissues were mixed well before total RNA isolation.

#### Target preparation and hybridization

Biotinylated and fragmented cRNA was prepared by the Affymetrix protocol and hybridized to Affymetrix GeneChip® Porcine Genome Array (each target organ from a single animal was profiled in duplicate; there were thus 4 replicate transcriptome datasets for each organ/tissue in each study group). Each array contained probe sets for 20,201 *Sus scrofa* genes and was scanned in a GeneChip® Scanner 3000 (Affymetrix Inc., Santa Clara, U S A).

#### Data analysis

We normalized signal intensities by global scaling to an arbitrary value of 500 using Microarray Suite software, version 5.0 (Affymetrix). We next analyzed normalized data by target organ or tissue type. Genes with absent calls in ≥10 out of 12 datasets (2 arrays×2 animals×3 groups) were excluded. We selected genes that were differentially expressed (i.e. ≥1.5-fold difference) in three pairwise comparisons (i.e. normal vs. untreated diabetic, normal vs. treated diabetic and treated diabetic vs. untreated diabetic) using FiRe v. 2.2 [31]. These genes, listed by log2 intensity values, were analyzed using Significance Analysis of Microarrays (SAM) version 1.21 based on 1,000 permutations [32]. Pairwise comparisons allowed us to obtain three gene sets, each having a false discovery rate <1%. The inter-relationship of these three gene sets was established by Venn diagrams (FiRe v. 2.2 software).

### Statistical Analysis

Data were expressed as mean±s.e.m. The number of animals is denoted by N and the total number of data points by n. Groups were compared using Student's unpaired two-sided *t* test (for data with equal variances) or Mann-Whitney *U*-test (for data with unequal variances). One-way ANOVA with Bonferroni correction was used for multiple group comparisons. All statistical tests and the area under the curve (AUC) were calculated with GraphPad Prism (GraphPad Software Inc., U S A). Cluster analysis of GeneChip® data was performed using algorithms in Genowiz (Ocimum Biosolutions Ltd., India). *P*<0.05 was considered significant.

## Results

### Regulated Insulin Production In Vitro

**Figure 1:**
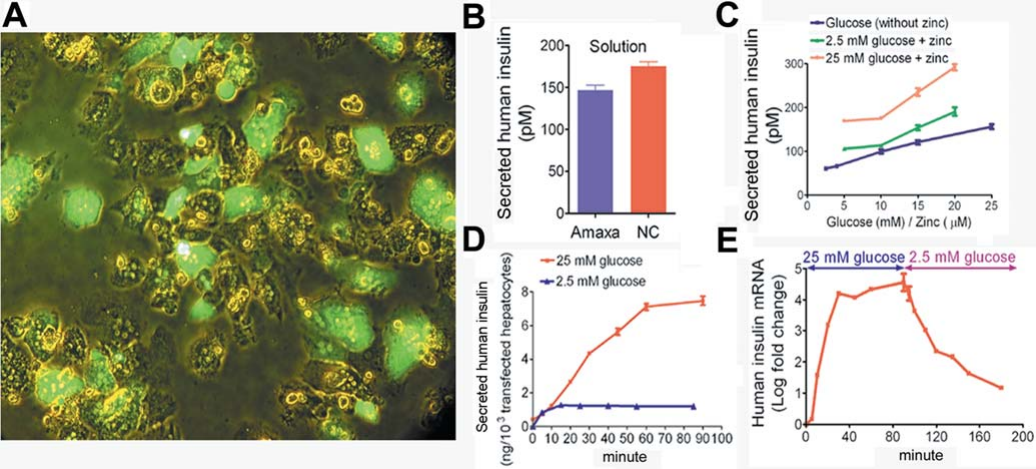
Regulated insulin secretion by electroporated primary porcine hepatocytes ex vivo. (A) Electroporation using Amaxa™ solution transfected 40–50% hepatocytes with pEGFP. Merged brightfield and fluorescence images; original magnification 200×. (B) Human insulin concentration in 24 h conditioned media of primary hepatocytes electroporated with p3MTChins (2 µg/10^6^ cells) in Amaxa or NC solutions (*p* = 0.0026). (C) Human insulin concentration in 24 h conditioned media of p3MTChins-electroporated hepatocytes. Thresholds for stimulating insulin secretion were 4 mM glucose (*p* = 0.0018 cf. 2.5 mM glucose) and 5 µM zinc (*p*<0.0001 cf. no zinc). Data are means±s.e.m. of quadruplicate experiments. (D) Time course of insulin secretion by electroporated hepatocytes. Insulin secretion increased from 0.01 to 0.14 ng/10^3^ hepatocytes/min (*p*<0.0001) after 5 min in 25 mM glucose but not in 2.5 mM glucose over 85 min. Data are means±s.e.m of quadruplicate experiments from 2 primary hepatocyte preparations. (E) Insulin mRNA, quantified by real time RT-PCR after subtracting a parallel minus-RT control and normalising to HPRT1 mRNA [19], rose within 5 min in 25 mM glucose (*p*<0.0001) and fell within 10 min in 2.5 mM glucose (*p* = 0.0149). Values are mean±s.e.m. of triplicate experiments.

The efficiency of electrogenetransfer in fresh porcine hepatocytes was 40–50% using a proprietary nucleofection solution (Figure 1A). We developed solution NC for electroporation and achieved transfection efficiencies of >50% with >80% viability, and consistently higher insulin secretion (Figure 1B). All experiments thereafter used solution NC. The glucose- and zinc-responsive promoter in p3MTChins was functional in porcine primary hepatocytes. Insulin secretion by electroporated hepatocytes in vitro was stimulated by glucose (4–25 mM) or zinc (5–20 µM) alone, with highest induction by glucose and zinc combined (Figure 1C). These glucose and zinc concentrations were within the physiological ranges in pig blood, 3.4–15.3 mM (J. Laukkarinen, unpublished data) and 13–20 µM [33], respectively. Insulin secretion rate rose from 0.01 to 0.14 ng/10^3^ hepatocytes/min (*p*<0.0001) from the 5th to 10th minute after increasing glucose from 2.5 to 25 mM while in a parallel incubation it remained stably low in 2.5 mM glucose (Figure 1D). Insulin transcription increased 1.5-fold within 5 min in 25 mM glucose, and decreased by about 8-fold within 10 min of restoring glucose concentration to 2.5 mM (Figure 1E).

### Irreversible Ablation of Endogenous β Cells in Streptozotocin-Diabetic Swine

**Figure 2:**
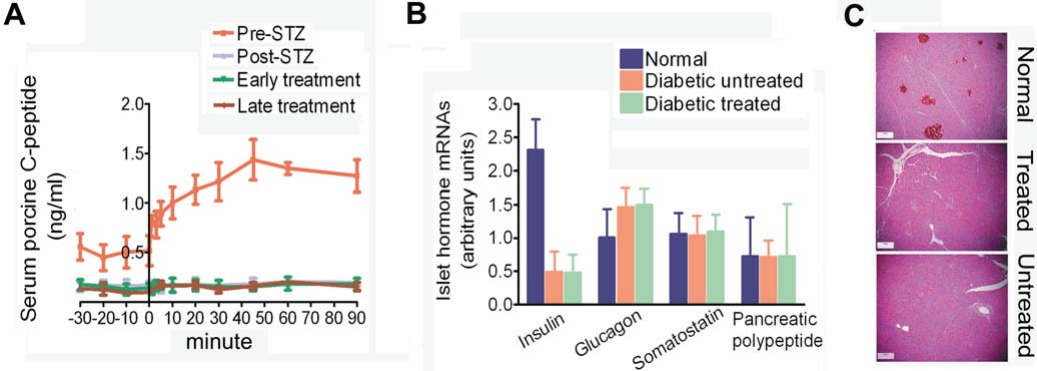
Absence of insulin expression in pancreatic β cells of STZ-diabetic pigs. (A) Persistent abolition of IVGTT-induced rise in serum PCP in the late treatment phase (13–47 wk after hepatocyte transplantation). Data are from 14 pigs. (B) Transcript analyses of islet hormones from pancreata of healthy, untreated diabetic (38–46 wk post-STZ) and treated diabetic (38–49 wk post-STZ) pigs (n = 2 per group) by real time RT-PCR as in Fig. 1E. Insulin mRNA was significantly decreased and glucagon mRNA significantly increased in both groups of diabetic pigs compared to healthy controls (*p*<0.0001 for all comparisons). Both diabetic groups had similar expression of insulin and glucagon (*p* = 0.4041 and 0.2308, respectively). Somatostatin and pancreatic polypeptide expression was not significantly different among all groups. (C) Insulin-positive cells in normal islets of a healthy pig cf. absent in diabetic pigs 38 wk post STZ.

**Figure 3:**
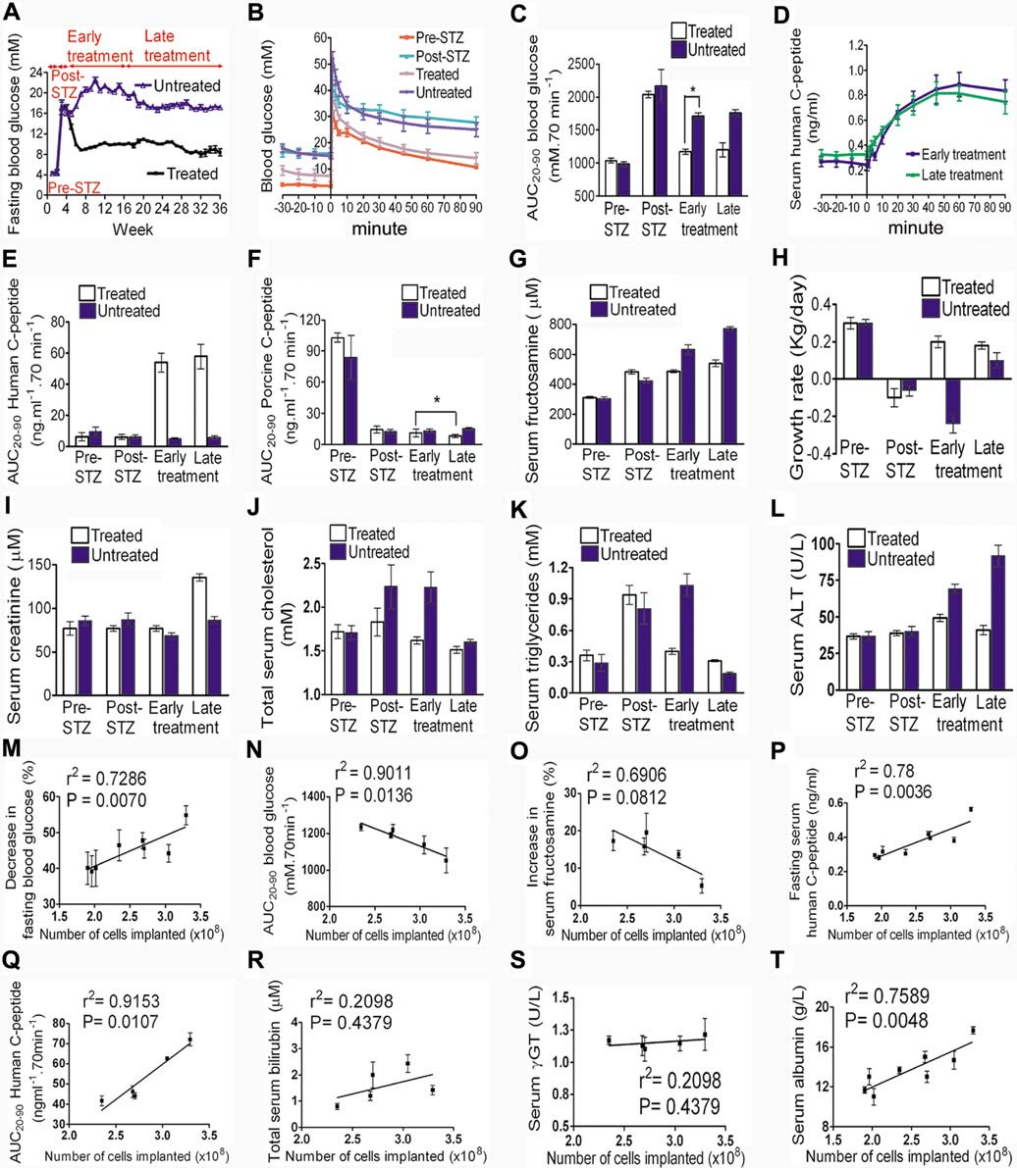
Glycemic and metabolic correction after implantation of insulin-secreting autologous hepatocytes. Aggregated data are presented from four sequential phases: pre-STZ (non-diabetic phase); post-STZ (≤10 d after STZ injection); early treatment (≤12 wk post-implantation) and late treatment (13–47 wk post-implantation) for 10 treated and 4 untreated pigs. See Supporting Table S1 for number of animals (N), separate analyses (n), means, s.e.m. and range for all biochemical tests performed. (A) Mean fasting glycemia in early and late treatment phases were significantly lower in treated pigs (*p*<0.0001 for both phases). (B) Glycemic profiles during IVGTT in the 4 study phases. Pre- and post-STZ profiles are pooled data from all pigs. Profiles of treated and untreated pigs were from the late treatment phase. (C) Glycemic data from panel (B) expressed as AUC_20–90BG_. Implanted insulin-secreting hepatocytes improved glucose tolerance (*, *p*<0.0001) with durable effect (*p* = 0.3481 early vs late treatment phase). (D) Serum HCP levels in treated pigs increased 5 min after intravenous glucose (*p* = 0.0124 and 0.0633 in the early and late treatment phases, respectively, cf. unstimulated HCP levels). (E) AUC_20–90HCP_ of treated pigs was comparable in both treatment phases (*p* = 0.8279). (F) AUC_20–90PCP_ confirmed β-cell ablation in STZ-diabetic pigs throughout the study (*p*<0.0001 both treatment phases cf. pre-STZ phase); *, *p* = 0.8279. (G) Mean serum fructosamine levels were lower in treated pigs in the early and late treatment phases (*p* = 0.0014 and <0.0001, respectively. cf. untreated pigs). Fructosamine levels did not rise significantly in treated pigs (*p* = 0.1500) but did increase among untreated pigs (*p* = 0.0015; early vs late treatment phase). (H) Body weight gain was higher among treated pigs (*p*<0.0001 early treatment and *p* = 0.0002 late treatment phase). (I) Mean serum creatinine concentration in treated pigs during the late treatment phase was significantly higher than untreated diabetics (*p*<0.0001). (J) Total serum cholesterol was higher in untreated pigs during the early treatment phase (*p* = 0.0035). Treated pigs maintained normocholesterolemia during the late treatment phase. Reduced serum cholesterol levels in untreated pigs during the late treatment phase were associated with marked weight loss. (K) Hypertriglyceridemia developed acutely in all pigs after onset of diabetes (*p* = 0.0002). It declined significantly in treated pigs during the early phase (*p*<0.0001 cf. untreated pigs). Marked reduction in serum triglyceride concentrations during the late treatment phase was associated with severe weight loss in untreated pigs. (L) Mean serum ALT activities in treated pigs during pre-STZ and late treatment phases were comparable (*p* = 0.8508) but were significantly higher in untreated pigs (*p*<0.0001 pre-STZ vs both treatment phases). Serum ALT decreased in treated pigs (early vs. late treatment values, *p* = 0.0228) but rose significantly in untreated pigs over the same period (*p* = 0.0086). (M–Q) The number of transplanted hepatocytes correlated with percentage decrease in fasting hyperglycemia, decrease in AUC_20–90BG_, stabilization of serum fructosamine, rise in fasting serum HCP levels and AUC_20–90HCP_. (R, S) Serum bilirubin and γ-glutamyl transpeptidase levels did not correlate significantly with the number of implanted hepatocytes but (T) serum albumin levels correlated positively.

Hyperglycemia developed within 48 hours of STZ administration and diabetes was stable for at least 49 wk. AUC_20–90PCP_ (an index of glucose-stimulated porcine insulin secretion) in the post-STZ and late treatment phases were not significantly different (*p* = 0.6394), evidence that endogenous β cells did not regenerate throughout the study (Figures 2A and 3F). Consistent with selective β cell ablation in STZ-diabetic pigs, insulin mRNA by RT-PCR was significantly decreased (*p*<0.0001; Figure 2B), somatostatin and pancreatic polypeptide transcript levels were unaltered, while glucagon mRNA showed the characteristic increase associated with diabetes (*p*<0.0001) [34] (Figure 2B). Pancreatic islets of diabetic swine did not stain for insulin unlike normal pig pancreas (Figure 2C).

### In Vivo Metabolic Effects After Implantation of Insulin-Secreting Autologous Hepatocytes

Fourteen diabetic pigs were implanted with autologous hepatocytes. In 10 pigs, hepatocytes were electroporated with p3MTChins (treated group) while hepatocytes of the remaining 4 pigs were mock electroporated without plasmid (untreated group). In vivo implantation data (Figure 3) were grouped into four temporally sequential phases: pre-STZ (non-diabetic; ≥4 wk), post-STZ (diabetic; 10 d), early and late treatment (≤12 and 13–47 wk, respectively, post-implantation). No animal received insulin injections in the early or late treatment phases.

#### Glycemic correction

Mean fasting glycemia was significantly lower in treated than in untreated pigs (*p*<0.0001) over the entire study period of at least 47 wk (Figure 3A. See Table S1 for details of Figure 3 and other biochemical data). Hypoglycemia (<2.8 mM) was not recorded in >320 separate fasting and random blood glucose measurements over this period. Glycemic profiles from IVGTT of treated animals were restored almost to their non-diabetic status (Figure 3B). IVGTT data were expressed as the area under the curve for blood glucose 20–90 min after intravenous glucose (AUC_20–90BG_), an integrated measure of in vivo glucose utilization. Among treated pigs, mean AUC_20–90BG_ decreased 74% from pre-implantation diabetic values, an effect that persisted into the late treatment phase when it was comparable to AUC_20–90BG _of the non-diabetic phase (*p* = 0.1348) (Figure 3C).

#### Regulated insulin secretion in vivo

Improved glucose tolerance was accompanied by near-normalization of the temporal profile of serum transgenic HCP during IVGTT. It was noteworthy that, similar to glucose-stimulated insulin secretion in vitro (Figure 1D), serum HCP levels of treated pigs rose within 5 min during IVGTT (Figure 3D), comparing favorably to the rise in serum PCP within 1 min of the same animals before diabetes induction (Figure 2A). Comparing IVGTT data of pre-STZ (serum PCP) and post-implantation (serum HCP) phases, these concentrations declined 40 min (0.0036 ng^−1^ ml^−1^ min^−1^) and 60 min (0.0020 ng^−1^ ml^−1^ min^−1^), respectively, after glucose stimulation. IVGTT data of treated diabetic pigs showed that the area under the curve for serum HCP 20–90 min after intravenous glucose (AUC_20–90HCP_), an integrated measure of human insulin secretion by implanted hepatocytes, was 54% that of AUC_20–90PCP_ (a comparable measure of endogenous insulin secretion from porcine pancreatic islets) of the same animals before induction of diabetes. Using species-specific C-peptide radioimmunoassays, glycemic improvement was attributed to human insulin secreted from transfected hepatocytes (Figure 3E) rather than recovery of endogenous β cells as AUC_20–90PCP_ values were not significantly different between treated and untreated groups, and were consistently <16% of pre-STZ values (Figure 3F).

#### Metabolic correction

As pig erythrocytes are impermeable to blood glucose [35], serum fructosamine was used instead as an index of average glycemic control. Mean fructosamine concentrations fell significantly from diabetic levels in treated pigs (*p* = 0.0014 early treatment; *p*<0.0001 late treatment). Unlike untreated diabetic controls, serum fructosamine did not rise significantly during the late treatment phase (*p* = 0.1500 cf. early treatment phase) (Figure 3G).

Emaciation is a known global metabolic effect of severe insulin deficiency. Our data showed that clinically effective restoration of insulin production was accompanied by a higher rate of body weight gain in treated animals (*p*<0.0001 early treatment; *p* = 0.0002 late treatment cf. untreated controls) (Figure 3H). Consistent with greater body weight gain, treated pigs had higher late treatment phase serum creatinine levels compared to untreated controls (*p*<0.0001) whose serum creatinine levels did not change throughout all study phases (Figure 3I). There was, however, no significant difference in blood urea and serum electrolyte concentrations between the groups (Table S1). Thus, higher serum creatinine levels were the result of increased muscle mass in treated animals rather than impaired renal function [36].

Dyslipidemia secondary to diabetes was also corrected. Among treated pigs, mean total serum cholesterol concentration was significantly reduced in the early treatment phase (*p* = 0.0035 cf. diabetic levels) while it remained unchanged in untreated animals (*p* = 0.9793) (Figure 3J). Hypertriglyceridemia developed acutely after induction of diabetes in both groups and significantly improved only in treated pigs during the early treatment phase (*p*<0.0001) (Figure 3K) and was not significantly different from the pre-STZ phase (*p* = 0.4935). However, serum cholesterol and triglyceride concentrations of untreated diabetic pigs also fell substantially during the late treatment phase (Figure 3J and 3K)–even to levels below their own non-diabetic values. This hypolipidemia associated with severe diabetes was a known effect of prolonged starvation and weight loss on lipoprotein metabolism [37].

High serum ALT activities are typical of liver dysfunction in poorly controlled diabetes and readily reverse after correction of hyperglycemia [38]. Mean serum ALT activities of treated animals during pre-STZ and late treatment phases were not significantly different (*p* = 0.8508) showing that treatment had effectively prevented diabetes-related liver dysfunction. In contrast, serum ALT activity rose progressively in untreated controls and were higher during the late treatment phase compared to both the early treatment and pre-STZ phases (*p* = 0.0086 and <0.0001, respectively) (Figure 3L).

#### Treatment outcomes correlated with number of hepatocytes implanted

Correction of disordered glucose metabolism by several objective measures-fasting glycemia (Figure 3M), intravenous glucose tolerance (Figure 3N) and serum fructosamine levels (Figure 3O)–was the result of restored insulin production shown by fasting serum HCP levels (Figure 3P) and AUC_20–90HCP_ (Figure 3Q). All these outcome measures correlated significantly with the number of hepatocytes implanted. In contrast, cell number did not correlate with serum total bilirubin concentrations (Figure 3R) or serum GGT activity (Figure 3S) showing that direct intrahepatic placement of hepatocytes, suspended in up to 15 ml, did not cause cholestasis. Overall restoration of normal liver functions in treated animals could be inferred from serum albumin trends. Serum albumin concentrations were positively and significantly correlated with the number of hepatocytes implanted (Figure 3T). They were also significantly lower in the late treatment phase of untreated animals compared to pre-STZ values (*p*<0.0001). In contrast, serum albumin concentrations in the late treatment phase of treated animals were not significantly different from the corresponding pre-STZ values (*p* = 0.1995).

### Engraftment of Insulin-Secreting Hepatocytes

Despite clear evidence of continuing transgenic insulin expression and metabolic correction in the treated animals, autopsies were performed on healthy controls, untreated diabetic and treated diabetic pigs 34–47 weeks post-implantation to systematically evaluate engraftment, vector persistence and target organ injury.

**Figure 4:**
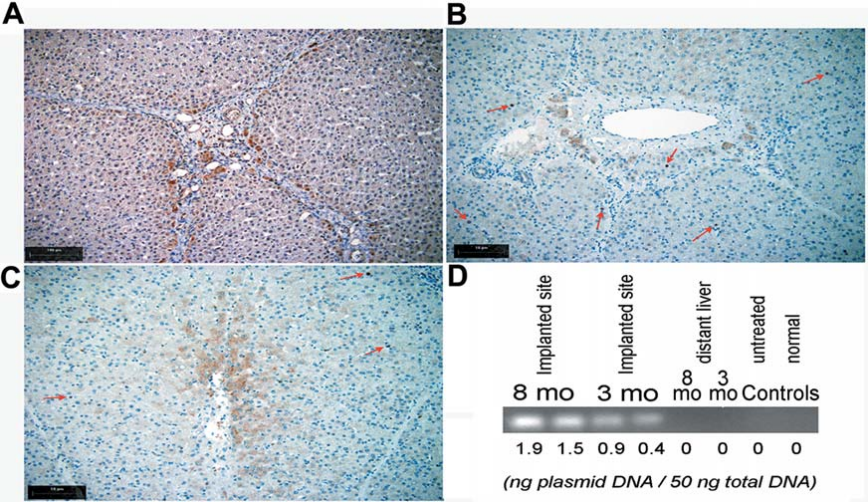
Engraftment of insulin-expressing autologous hepatocytes. Immunostained liver 36 wk after hepatocyte transplantation showed normal lobular architecture and insulin-producing hepatocytes engrafted around the portal triads (A) and central veins (B). Panels (B) and (C) show co-staining for insulin and mitotic markers, PCNA and Ki67, respectively. Mitotically positive cells were rare (arrowed). (D) Quantitative PCR using vector-specific primers detected p3MTChins in liver 3 and 8 mo after implantation. The amount of vector detected in each sample is expressed in ng plasmid DNA below the panel.

Autologous insulin-secreting hepatocytes had engrafted mainly, but not only, around portal triads and central veins (Figure 4A–C). There were no features of disordered lobular architecture or cirrhosis in the implanted livers. No tumors were detected during careful examination of the liver, spleen, intestinal tract, peritoneal cavity, kidneys, adrenals, lungs and heart. Fasting serum HCP levels during the first week after implantation (0.44±0.03 ng/ml) stabilized at 0.31±0.02 ng/ml after 4 weeks, consistent with functional engraftment of 70% of implanted hepatocytes. Concordant with persistent secretion of transgenic insulin (Figure 3E), plasmid DNA was detected 3 and 8 months post-implantation using vector-specific primers for PCR of total DNA extracted from thick sections of liver that showed insulin-producing hepatocytes (Figure 4D). In contrast, vector DNA was barely detectable in the same liver lobes distant from implantation sites.

Physiological concentrations of insulin are mitogenic [39] and liver tumors have been reported in all diabetic animals intrahepatically transplanted with low numbers of pancreatic islets [40]. However we showed that, compared to liver from a healthy control, the liver of implanted animals did not show increased mitogenic activity as assessed by immunostaining for proliferating cell nuclear antigen (PCNA) and Ki67 (Figures 4B and C). Rare PCNA and Ki67 staining was observed in both insulin-secreting as well as adjacent hepatocytes, indicating that ectopic insulin secretion in the liver did not have autocrine or paracrine mitogenic effects. Insulin-secreting hepatocytes were morphologically normal without features of cellular transformation.

### Treatment Attenuated Target Organ Injury

Tissues obtained from systematic autopsies performed on sex- and age-matched normal healthy Yorkshire pigs, untreated diabetic and treated diabetic pigs (2 animals per group) were evaluated to ascertain effects of treatment on diabetes-induced target organ injury. To allow sufficient time for development of secondary micro- and macrovascular complications, both groups of diabetic animals were maintained for 34–42 weeks before autopsy.

#### Treatment effects on tissue structure

**Figure 5:**
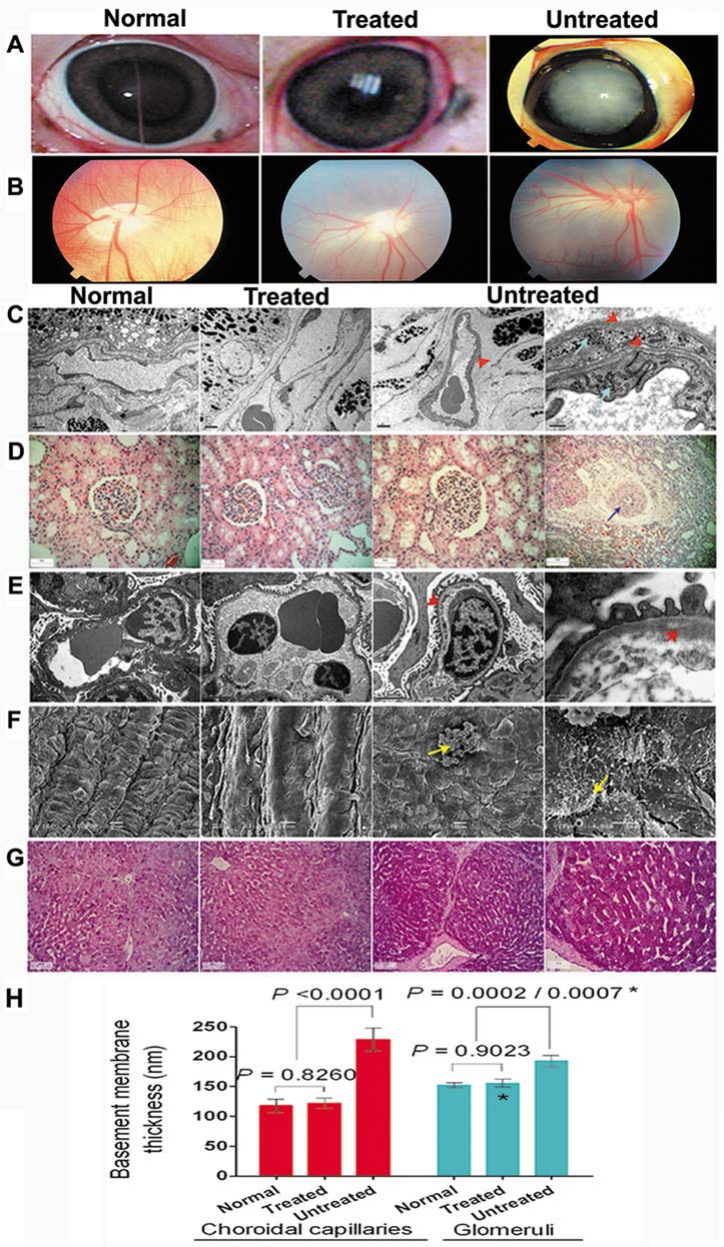
Treatment attenuated target organ injury. (A) Example of a dense cataract observed only in untreated diabetic pigs. (B) Retinal fluorescein angiography showed normal capillary permeability. (C) TEM of retina showed thickened basement membrane of a choroidal capillary, duplicated basement membrane (red arrow) and an extra layer of cells in the capillary wall (blue arrow). (D) H&E stained kidney sections showed enlarged glomeruli with mesangial expansion, and arteriolar wall thickening (arrow) in untreated diabetes. (E) TEM of untreated diabetic kidney showed thickening of glomerular basement membrane. (F) SEM showed abnormal endothelial surface structures only in carotid arteries of untreated diabetic pigs. (G) Increased intensity of PAS staining in untreated diabetic liver without fibrosis or cirrhosis. (H) Basement membrane thickness, measured in 20 choroidal capillaries and 20 renal glomeruli of each group (n = 2), was significantly greater in untreated diabetes cf. both treated and healthy animals. *, treated vs. untreated.

Both untreated diabetic pigs had dense posterior subcapsular cataracts but not both treated pigs (Figure 5A). The choroidal capillary basement membrane in the retinae of untreated diabetic pigs was thicker than that of both healthy and treated diabetic animals (Figure 5H; *p*<0.0001 for both comparisons). In contrast, there was no difference between capillaries of healthy and treated diabetic animals (*p* = 0.8260). Another striking feature of choroidal capillaries in untreated diabetes was the presence of an additional cell layer invested with its own basement membrane (Figure 5C). Retinal capillary permeability was normal by fluorescein angiography (Figure 5B).

Kidneys of untreated diabetic pigs showed mesangial hyperplasia and arteriolar thickening, characteristics of diabetic nephropathy that were absent in healthy and treated diabetic animals (Figure 5D). Glomerular basement membranes were thicker in untreated diabetic pigs compared to healthy (*p* = 0.0002) and treated diabetic pigs (*p* = 0.0007) (Figures 5E and G). Notably, glomerular basement membrane thickness of treated diabetic pigs was not different from healthy controls (*p* = 0.9023).

Scanning electron microscopy of carotid arteries from an untreated diabetic pig showed a smoothened endothelial surface, abnormal microvilli and clumped erythrocytes with fibrin adhering to the endothelium while carotid arteries of a treated diabetic pig appeared more similar to a healthy animal (Figure 5F).

Hepatocytes of untreated diabetic pigs stained more intensely with periodic acid-Schiff stain (Figure 5G) indicative of glycogenic hepatopathy, a known but less well-recognized complication of diabetes [38].

#### Treatment effects on target organ transcriptomes

**Figure 6:**
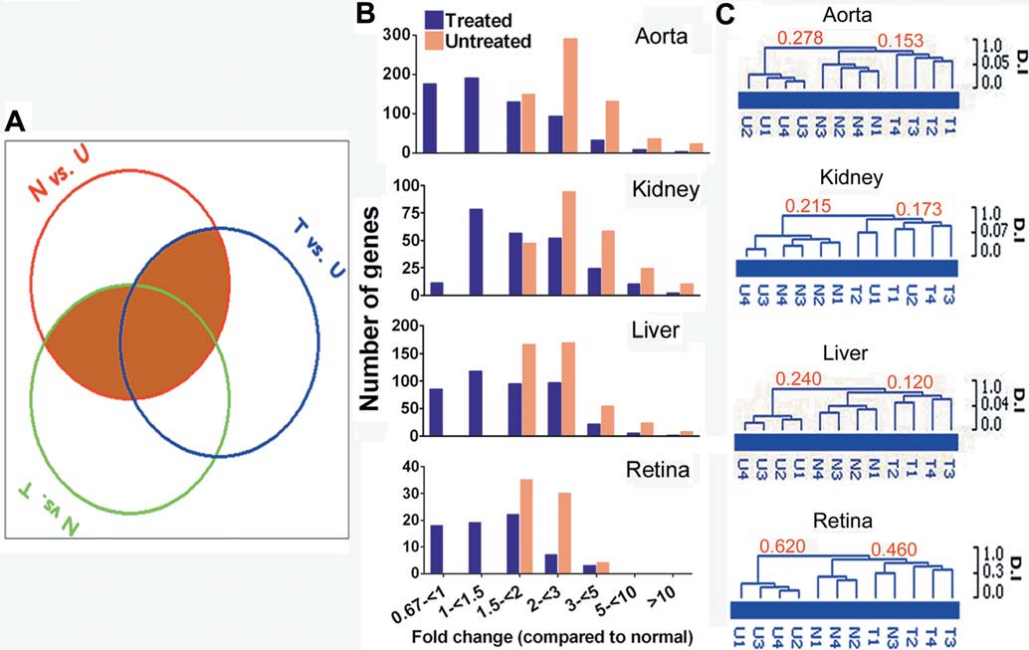
Transcriptional profiling of aorta, kidney, liver and retina. (A) Venn diagram of genes whose expression was significantly different in pairwise comparisons: N vs U; N vs. T and T vs. U (N, healthy controls; T, treated diabetic; U, untreated diabetic pigs. n = 2 per group). Genes ≥1.5-fold different in expression in pairwise comparisons were analysed by SAM (significance analysis of microarray) based on 1,000 permutations (false discovery rate <1%). Shaded areas denote diabetes-affected genes. (B) Expression of diabetes-affected genes in untreated and treated pigs grouped in increasing order of differential expression relative to healthy pigs. Genes represented by multiple probe sets were merged and averaged. (C) Unsupervised hierarchical clustering of diabetes-affected genes showed a distinct tendency for tissue transcriptomes of treated diabetic and healthy pigs to cluster together. Replicate transcriptional profiles of the same animal are designated 1 and 2, or 3 and 4 (i.e. 2 pairs of transcription datasets per group of 2 animals). Numbers on selected nodes of each dendrogram are the dissimilarity index (D.I.).

Transcriptional changes are sensitive molecular sentinels of the injurious effects of diabetes on organs targeted for developing secondary complications [41] e.g. renal failure, blindness and vascular insufficiency. Retina, kidney, aorta and liver of healthy normal pigs, untreated diabetic and treated diabetic pigs were flash frozen in liquid nitrogen and transcriptionally profiled because these organs are known targets for the microvascular, macrovascular and metabolic complications of diabetes. We compared each tissue transcriptome among the three animal groups. Of 20,201 genes represented on the microarray, subsets of 625, 233, 419 and 69 genes were identified as diabetes-associated in aorta, kidney, liver and retina, respectively, using two filters of significance (Figure 6A). Among the most highly dysregulated diabetes-associated genes were protein kinase C and vascular cell adhesion molecule in aorta, retinol-binding protein and angiopoietin-like protein in kidney, lipoprotein lipase and apolipoprotein A-IV in liver, vasoactive intestinal peptide receptor and serpin peptidase inhibitor in retina. Implantation of insulin-secreting autologous hepatocytes in diabetic pigs corrected dysregulated expression of 58% (363 of 625), 38% (89 of 233), 48% (203 of 419) and 54% (37 of 69) genes in aorta, kidney, liver and retina, respectively (Figure 6B). Unsupervised clustering of diabetes-associated genes in each tissue transcriptome showed that treated diabetic pigs generally clustered with healthy controls than with untreated diabetic pigs (Figure 6C).

## Discussion

This study demonstrates that autologous primary adult hepatocytes of a large preclinical animal can be efficiently, simply and safely electroporated ex vivo with a nonviral vector to physiologically express, process and secrete insulin in vivo with substantial therapeutic efficacy for at least 47 weeks. Furthermore, we provide evidence that such treatment attenuated tissue injury secondary to diabetes i.e. metabolic, micro- and macrovascular complications. In addition, our data indicate a ‘dosing effect’ i.e. glycemic correction, glucose tolerance and human C-peptide secretion were each correlated significantly with the number of implanted hepatocytes. Our data showing that significant metabolic correction was achieved by transfected hepatocytes whose glucose-stimulated insulin secretion exceeded 50% that of endogenous β cells is in agreement with maintenance of normoglycemia in human subjects after 50% pancreatectomy [42]. The number of autologous hepatocytes we implanted was about 10-fold lower than the number of allogeneic donor hepatocytes that partially corrected inherited factor VII deficiency in two children [43].

Use of a porcine model that resembles humans in size, anatomy, physiology and pathophysiology has moved our approach closer to clinical application. Despite many past attempts at developing gene- and autologous cell-based diabetes treatments, none of the approaches that are effective in murine diabetes has been successfully adapted to large animals, let alone human subjects. This longstanding impasse highlights the problem of scalability, among others that continue to impede clinical development of genetic and cellular therapies [18], [44]. Allogeneic whole pancreas and isolated islet transplantation have a proven record of success in well-selected diabetic patients [21], [22], [45]. It is evident, however, that these procedures have a restricted impact in diabetes care because of donor scarcity, specialised skills required and high treatment costs. By using autologous hepatocytes, our approach is not donor dependent, technically facile and does not require long-term immunosuppression. Rodent studies suggest that insulin-expressing hepatocytes may be protected from the anti-beta cell specific immune response underlying autoimmune diabetes [23].

In this study, we chose to isolate hepatocytes from surgically accessible autologous liver that was capable of yielding hepatocytes in adequate number and quality in a planned procedure. The amount of excised tissue (less than 10% of the liver) was well within the regenerative capacity of the liver to restore [47]. We chose to reimplant hepatocytes directly into the liver parenchyma instead of via the portal vein, as practised by most investigators, because more than 70% of portal vein-infused hepatocytes were trapped within the liver vasculature and quickly destroyed by blood macrophages, leaving only a small fraction to engraft successfully [48] while up to 50% were distributed to extrahepatic organs [49]. Portal infusion of hepatocytes in adult pigs was associated with intraportal thrombosis and pulmonary embolism with infarction [50]. In contrast, hepatocytes implanted directly into the liver parenchyma did not distribute to other organs and integrated normally into liver lobules [51]. Here we show that intraparenchymal hepatocyte implantation achieved significant engraftment, did not disrupt normal hepatic lobular architecture, did not alter normal biochemical indices of liver function, was safely performed percutaneously under ultrasonic guidance with comparable results to open implantation and thus was not inferior to portal venous infusion of cells. A single surgical death from intraabdominal hemorrhage due to iatrogenic liver laceration occurred during percutaneous implantation. This animal was excluded from analysis. There was no surgical mortality and morbidity after we adopted the reverse Trendelenburg position for percutaneous hepatocyte implantation.

We chose to test the feasibility of this approach in a pre-clinical diabetes model for several reasons. Deficiency of a single circulating protein i.e. insulin underlies type 1 diabetes and is a significant abnormality in the later stages of type 2 diabetes. Despite multiple treatment methods, a lifetime of euglycemic control is achievable only with intensive efforts that are difficult to sustain. Hepatocytes are suitable candidates for developing insulin-secreting bioimplants because of their proven physiological glucose-sensing and glucose-responsive characteristics, and proficiency in protein biosynthesis and secretion. Portal venous drainage makes the liver a prime anatomic location for nutrient-regulated insulin secretion. Although hepatocytes lack secretory granules, we showed dynamic changes in insulin secretion in tandem with changes in extracellular glucose concentrations that were sufficiently rapid to be near-physiological in onset and offset. Consequently, hypoglycemia was not recorded or clinically observed even after 16-hour fasts. This is noteworthy as current clinical practice aimed at achieving tight glycemic control often increases the risk of hypoglycemia. For instance, diabetic children on conventional insulin therapy sustain about 20 hypoglycemic comas and convulsions per 100 patient years [52].

Therapeutic efficacy in this study extended beyond glycemic and metabolic (including dyslipidemia) correction to protection against complications secondary to tissue injury. Our data provide evidence that early and effective restoration of normal metabolism in diabetes prevents microvascular and macrovascular abnormalities in the eye, kidney and large vessels [53], as well as diabetic hepatopathy [38]. Basement membrane thickening was more severe in choroidal capillaries than in renal glomeruli probably because choroidal capillaries have much higher blood flow rate [54], and suggests that ocular tissue injury from ischemia is a likely early complication of poorly controlled diabetes. These findings advocate more aggressive clinical management to attain and maintain normoglycemia early in the course of diabetes as effective protection against serious target organ injury.

Other salient features of our approach contributed to the outcome of this study. Because we chose to use non-tumorigenic primary and autologous cells that are resistant to most conventional techniques of gene transfer, we resorted to an ex vivo approach using electrical pulsing conditions that would cause severe tissue necrosis, if administered in vivo. Avoiding primary cultures was possible because we did not aim to transdifferentiate hepatocytes into β-like cells and our electroporation technique achieved high transfection efficicencies (>50% with >80% viability). This also obviated the risk of inducing adverse genomic instability known to occur when primary somatic cells are cultured in vitro [26], [27]. Ex vivo electroporation of primary hepatocytes with nonviral vectors is likely to be more clinically acceptable than viral transduction for its comparable efficiency and simplicity [55]. Ex vivo transduction of primary hepatocytes has the attendant risk of co-implanting free viral particles with attendant risks of adverse immunogenic or genotoxic effects. Compared to viral vectors, pharmaceutical grade plasmid vectors are simpler and less costly to produce, have low toxicity and immunogenicity, and are non-pathogenic. Histological examination shows that plasmid-electroporated hepatocytes were morphologically normal without features of cellular transformation. This showed that electroporation of hepatocytes with a nonviral vector was safe i.e. was not oncogenic or mutagenic. Indeed, mutations resulting from rare plasmid integrations after electroporation are estimated to be at least three orders of magnitude lower than the background frequency of spontaneous genome mutations [56]. Although short-term transgene expression is generally expected from non-integrating vectors, several reasons explain the surprisingly sustained therapeutic effects we achieved namely, physiological mammalian promoters, unlike viral promoters, are less susceptible to silencing [57], electroporation transfers vectors in high copy number [58] and non-mitotic hepatocytes durably retain plasmid vectors [55].

We recognize that our approach could have greater clinical appeal if the method for procuring autologous primary hepatocytes were less invasive. In this regard, it may be possible to employ laparoscopic resection of an even smaller liver wedge (currently about 10% of total liver mass) in future. This adaptation will require hepatocyte isolation in higher yield and greater transfection efficiency without compromising cell viability.

In conclusion, our approach simultaneously addresses several major difficulties in the field of cellular therapy i.e. donor scarcity, requirement for specialized and costly skills, complications of chronic immunosuppression, tumorigenicity of cell implants derived and differentiated from embryonic stem cells, immuno- and genotoxicity of viral vectors as well as transient transgene expression. Moreover, it warrants consideration for clinical development beyond diabetes because it has broad potential application for treating other acquired and inherited diseases for which systemic reconstitution of a specific protein deficiency is critical and could be especially facile for diseases that do not demand tightly regulated transgene expression.

## Supporting Information

Table S1 Biochemical data
(0.11 MB PDF)
