# Supplementary material for: Nonvirally Modified Autologous Primary Hepatocytes Correct Diabetes and Prevent Target Organ Injury in a Large Preclinical Model
Source: PLoS One. 2008 Mar 5;3(3):e1734. doi: 10.1371/journal.pone.0001734 (PMC2249706; doi:10.1371/journal.pone.0001734)
Supplement: Table S1 — Biochemical data (0.11 MB PDF) [file pone.0001734.s001.pdf]

Supporting table 1: Biochemical data

	Treated Animals (N=10)				Untreated Animals (N=4)			
Indices monitored	Pre-STZ	Post-STZ	Early treatment	Late treatment	Pre-STZ	Post-STZ	Early treatment	Late treatment
Fasting blood glucose (mM)	4.4 ± 0.3 (2.5-12.4) (n=59)	17.3 ± 0.6 (4.7->33.3) (n=97)	10.0 ± 0.2 (3.9-16.4) (n=178)	9.8 ± 0.4 (2.9-21.3) (n=144)	4.4 ± 0.5 (2.7-13.1) (n=27)	16.9 ± 1.2 (3.9->33.3) (n=32)	20.1 ± 0.5 (10.7->33.3) (n=93)	17.5 ± 0.4 (10.4->33.3) (n=94)
Exogenous insulin dose (U/kg/day)	Nil	0.46 ± 0.05 (0.30-0.71) (n=10)	Nil	Nil	Nil	0.27 ± 0.06 (0.09-0.35) (n=4)	Nil	Nil
Growth rate (kg/day)	0.30 ± 0.03 (0.15-0.38) (n=9)	-0.10 ± 0.05 (-0.27)-0.05 (n=9)	0.20 ± 0.03 (0-0.31) (n=9)	0.18 ± 0.02 (0.14-0.21) (n=3)	0.30 ± 0.02 (0.26-0.34) (n=4)	-0.06 ± 0.03 (-0.14)-0.01 (n=4)	-0.24 ± 0.15 (-0.59)-0.02 (n=4)	0.10 ± 0.04 (0.08-0.13) (n=2)
Fructosamine (μM)	311.9 ± 7.6 (282-363) (n=13)	483.1 ± 13.0 (414-560) (n=14)	486.9 ± 10.1 (313-645) (n=47)	538.8 ± 22.7 (350-766) (n=33)	305.8 ± 9.5 (276-365) (n=8)	424.6 ± 16.6 (352-488) (n=8)	632.2 ± 31.9 (362-885) (n=28)	771.4 ± 13.2 (578-895) (n=29)
Urea (mM)	5.67 ± 0.66 (2.1-9.1) (n=13)	3.81 ± 0.38 (2.0-8.0) (n=18)	5.16 ± 0.32 (2.2-13.4) (n=51)	4.90 ± 0.34 (2.1-8.0) (n=32)	5.53 ± 1.08 (2.2-9.7) (n=8)	6.64 ± 0.89 (4.6-10.0) (n=8)	6.74 ± 0.25 (4.4-10.3) (n=29)	4.88 ± 0.22 (1.0-6.7) (n=26)
Creatinine (μM)	77.2 ± 8.0 (52-156) (n=14)	77.3 ± 3.2 (52-104) (n=20)	77.1 ± 3.1 (25-121) (n=50)	135.3 ± 4.4 (91-187) (n=34)	85.9 ± 5.7 (57-101) (n=8)	87.3 ± 7.9 (58-126) (n=9)	68.5 ± 3.4 (25-101) (n=30)	86.4 ± 4.1 (65-161) (n=29)
Potassium (mM)	4.11 ± 0.12 (3.4-4.6) (n=12)	4.26 ± 0.09 (3.7-4.8) (n=18)	4.06 ± 0.05 (3.4-4.8) (n=48)	3.91 ± 0.06 (3.4-5.2) (n=33)	4.19 ± 0.16 (3.6-4.7) (n=8)	4.17 ± 0.12 (3.6-4.6) (n=9)	4.06 ± 0.06 (3.3-5.1) (n=32)	3.94 ± 0.06 (3.4-4.9) (n=26)
Total protein (g/L)	51.3 ± 1.1 (44-60) (n=20)	53.0 ± 1.1 (47-69) (n=29)	57.4 ± 0.8 (42-70) (n=52)	62.4 ± 1.2 (51-83) (n=35)	59.3 ± 2.6 (49-73) (n=8)	53.9 ± 1.5 (44-60) (n=9)	58.7 ± 1.3 (45-70) (n=30)	60.9 ± 0.9 (54-70) (n=24)
Albumin (g/L)	14.9 ± 0.4 (11-18) (n=21)	14.9 ± 0.3 (11-18) (n=30)	14.6 ± 0.3 (11-18) (n=52)	13.9 ± 0.5 (10-18) (n=35)	16.8 ± 1.1 (10-21) (n=8)	16.3 ± 0.7 (12-19) (n=9)	14.9 ± 0.3 (12-17) (n=30)	13.6 ± 0.3 (11-15) (n=24)
Total bilirubin (μM)	2.10 ± 0.25 (1.0-6.0) (n=21)	2.69 ± 0.27 (1.0-7.0) (n=30)	3.60 ± 0.50 (1.0-26.0) (n=53)	3.50± 0.41 (1.0-11.0) (n=35)	1.63 ± 0.18 (1.0-2.0) (n=8)	3.0 ± 0.65 (1.0-7.0) (n=8)	5.97 ± 0.96 (1.0-20.0) (n=32)	3.36 ± 0.42 (1.0-8.0) (n=24)
Alkaline phosphatase (U/L)	179.9 ± 9.3 (131-275) (n=21)	174.3 ± 10.0 (81-269) (n=30)	146.7 ± 6.3 (61-263) (n=54)	158.4 ± 8.4 (61-259) (n=35)	156.3 ± 10.0 (126-209) (n=8)	123.6 ± 13.9 (63-180) (n=9)	124.7 ± 10.7 (61-268) (n=32)	200.2 ± 7.4 (116-246) (n=24)
ALT (U/L)	36.9 ± 2.0 (27-59) (n=21)	39.0 ± 1.8 (24-57) (n=30)	49.4 ± 2.5 (25-135) (n=54)	41.0 ± 3.1 (15-96) (n=35)	36.9 ± 3.3 (25-48) (n=8)	40.2 ± 3.5 (31-65) (n=9)	68.9 ± 3.3 (43-147) (n=31)	91.6 ± 7.4 (34-183) (n=24)
γGT (U/L)	22.2 ± 2.0 (15-45) (n=21)	25.8 ± 2.6 (13-57) (n=30)	28.4 ± 1.9 (14-67) (n=54)	24.7 ± 0.7 (19-35) (n=35)	19.6 ± 0.7 (17-22) (n=8)	18.8 ± 1.0 (14-23) (n=9)	24.6 ± 1.2 (17-39) (n=32)	26.7 ± 0.7 (21-33) (n=24)
Triglyceride (mM)	0.36 ± 0.05 (0.15-0.88) (n=19)	0.94 ± 0.09 (0.39-1.87) (n=29)	0.40 ± 0.03 (0.17-1.29) (n=52)	0.31 ± 0.01 (0.14-0.38) (n=35)	0.29 ± 0.08 (0.12-0.83) (n=8)	0.81 ± 0.15 (0.33-1.61) (n=9)	1.03 ± 0.11 (0.17-2.26) (n=29)	0.19 ± 0.01 (0.11-0.31) (n=24)
Total cholesterol (mM)	1.72 ± 0.08 (1.25-2.80) (n=21)	1.83 ± 0.06 (0.86-2.36) (n=30)	1.62 ± 0.04 (1.11-2.38) (n=54)	1.51 ± 0.04 (1.14-2.03) (n=35)	1.71 ± 0.08 (1.35-2.01) (n=8)	2.23 ± 0.25 (1.70-3.78) (n=8)	2.22 ± 0.18 (1.37-5.41) (n=32)	1.60 ± 0.03 (1.31-1.80) (n=24)

HDL (mM)	0.92 ± 0.03 (0.64-1.22) (n=20)	1.02 ± 0.03 (0.53-1.27) (n=29)	0.91 ± 0.02 (0.51-1.17) (n=52)	0.91 ± 0.03 (0.61-1.18) (n=35)	0.91 ± 0.06 (0.71-1.21) (n=8)	1.34 ± 0.15 (0.58-1.72) (n=9)	1.14 ± 0.09 (0.55-2.16) (n=30)	0.97 ± 0.02 (0.73-1.13) (n=24)
AUC_{20-90} (BG) ($mM70min^{-1}$)	1036 ± 37 (941-1164) (n=5)	2036± 47 (1897-2121) (n=5)	1169 ± 41 (1136-1225) (n=5)	1201 ± 112 (1139-1256) (n=3)	986 ± 31 (913-1055) (n=4)	2164 ± 251 (1677-2300) (n=4)	1714 ± 48 (1490-1936) (n=4)	1760 ± 53 (1603-1898) (n=2)
AUC_{20-90} (HCP) ($ngml^{-1}70^{-1}$)	6.26 ± 2.56 (0-14.66) (n=5)	6.03 ± 1.65 (0-9.20) (n=5)	53.87 ± 6.18 (39.07-78.02) (n=5)	57.87 ± 7.90 (42.21-76.18) (n=3)	9.46 ± 3.08 (3.88-18.26) (n=4)	6.29 ± 1.12 (3.79-9.10) (n=4)	5.17 ± 0.61 (3.74-8.55) (n=4)	5.86 ± 0.86 (4.02-8.40) (n=2)
AUC_{20-90} (PCP) ($ngml^{-1}70^{-1}$)	102.92 ± 4.34 (91.96-104.31) (n=5)	14.34 ± 3.09 (8.77-23.64) (n=5)	11.10±3.56 (3.32-29.29) (n=5)	8.30±1.55 (6.02-13.19) (n=3)	83.67 ± 19.63 (57.42-117.89) (n=4)	12.48 ± 1.87 (8.52-16.20) (n=4)	13.02 ± 1.59 (8.55-16.81) (n=4)	15.11 ± 1.27 (11.49-18.26) (n=2)
